# Platform Leadership and Sustainable Competitive Advantage: The Mediating Role of Ambidextrous Learning

**DOI:** 10.3389/fpsyg.2022.836241

**Published:** 2022-02-24

**Authors:** Xiao Yang, Rong Jin, Changyi Zhao

**Affiliations:** ^1^School of Management, Jilin University, Changchun, China; ^2^School of Public Administration, Jilin University, Changchun, China; ^3^School of Business, Sichuan University, Chengdu, China

**Keywords:** sustainable competitive advantage, platform leadership, ambidextrous learning, exploratory learning, exploitative learning

## Abstract

In the context of the knowledge economy, the role of traditional leadership for enterprises is questioned. Based on contingency theory and the resource-based view, this paper proposes the important role of platform leadership, a new leadership type in line with the context of the times, for a sustainable competitive advantage. We conducted an empirical study to examine and confirm the positive effects of platform leadership on sustainable competitive advantage and ambidextrous learning. We also verified the mediation effect of exploratory and exploitative learning on platform leadership and sustainable competitive advantage. Additionally, relevant discussion and research contributions are put forward.

## Introduction

With the accelerating process of economic globalization, advanced technology and productivity are rapidly flowing worldwide, and new market opportunities are emerging. Meanwhile, competition among enterprises is becoming increasingly intense. How to gain a sustainable competitive advantage to meet the increasingly fierce competition in the market has become a common topic of concern for both the academia and industry. Studies have shown that the establishment of a sustainable competitive advantage increases the difficulty and cost for competitors to copy a company’s successful model (Barney, 1991). It also provides the material basis and strategic options for the company to develop new resources and markets and ensures that a strong competitive position and a competitive advantage are maintained in a long period (Benner and Tushman, 2003). Therefore, to respond to the competitive demands and achieve strategic goals, companies must pay attention to the construction of sustainable competitive advantage. This perspective is also widely accepted in academic circles (Asimakopoulos et al., 2020; Khouroh et al., 2020; Knudsen et al., 2021; Prabowo et al., 2021; Yang et al., 2021).

Leadership is an important role that influences others to make sustained efforts to achieve goals, and it is widely present at all levels of government, business, schools, hospitals, military, and social groups (Hao, 2016). For centuries, scholars have extensively discussed the topic of leadership, the relationship between leadership types and organizational development, business performance, and so on (Shamir et al., 1993; Bass, 1995; Karim et al., 2016). A mainstream study is to view leadership as a top-down hierarchical influence process, in which leaders play an irreplaceable role and have a significant impact on the firm or organization (Slater and Narver, 1995; Song and Long, 2017). In the 20th century, the era of industrial economy, the limited speed of change in the external environment and the level of employee knowledge prompted companies to consider specialization and division of labor in pursuing economic benefit. Therefore, pyramidal hierarchical structure with authoritative authority as the core was widely distributed in all types of organizations and brought better organizational effectiveness (Fletcher, 2004). Thus, there have been many discussions on the characteristics or behavioral characteristics of effective leadership. Moreover, the important role of different leadership characteristics, such as transformational leadership (Bass, 1995) and charismatic leadership (Shamir et al., 1993), on organizations has been recognized.

Since entering the 21st century, with the rapid development of information technology and network technology, the era of the industrial economy is rapidly changing to the era of knowledge economy, and the organizational environment has become increasingly dynamic, uncertain, and unpredictable. Organizations must learn continuously to adapt to the complex external environment, and flat organizational forms that can quickly transfer information and respond to competitive needs are gradually favored by enterprises (Meisel and Fearon, 1999; Yang et al., 2004). In the context of the dynamic organizational environment, the original hierarchical concepts in organizations are gradually weakened, and decentralization and de-leadership have become important trends. Moreover, the traditional top-down leadership model is beginning to be considered unsuitable for meeting the adaptive challenges of the knowledge economy (Uhl-Bien et al., 2007). The role of traditional leadership in organizational development has been seriously questioned (Avolio et al., 2009).

However, the advent of the knowledge economy does not mean that leadership is no longer important; rather, it places new demands on leaders and leadership models. Leaders should no longer think of themselves as the highest point of the pyramid, but rather be rooted at the grassroots level, focusing on employees and organizational development (Feng et al., 2014). The top-down hierarchical influence in organizations is weakening. However, leaders play a more important role in using a bottom-up approach to lead employees, respond to changes in the external environment, and facilitate the achievement of organizational goals (Morris et al., 2005). Based on the bottom-up leadership view, a series of meaningful studies have been conducted in the academic circle, and many new leadership types have been proposed and developed, such as humble leadership (Owens and Hekman, 2012) and inclusive leadership (Carmeli et al., 2010).

Other scholars have pointed out that considering subordinates’ development as the endpoint is still an idealized view (Hao et al., 2021). The more common reality is that the relationship between employees, leaders, and organizations is symbiotic and co-prosperous, and the development of employees usually depends on the development and growth of the organization (Hao et al., 2021). Based on this, scholars proposed the concept of platform leadership, which is used to explain the new model of effective leadership behavior characteristics under the background of a dynamic organization and the rise of knowledge workers. Hao (2016) specified that apart from motivating employees’ potential in a bottom-up manner, leaders should continuously focus on organizational development and make the organization a platform for the aggregation and integration of resources from all parties and the realization of value. Simultaneously, the platform needs to be continuously optimized and improved to cope with the dynamic changing environment faced by the organization and obtain or generate more high-quality resources through the joint growth of the organization, leaders, and employees, which is called platform leadership. Compared to other leadership traits, platform leadership emphasizes the interactive relationship between leaders, employees, and organization, and it may have a more intuitive impact on organizational learning and capacity.

In summary, the relationship between leadership, organizational development, and the competitive advantage shows great differences at different times. In today’s highly competitive globalization and rapidly developing knowledge-based economy, enterprises’ formation of a sustainable competitive advantage relies more on constructing internal knowledge management capabilities and controlling the external competitive environment (Liao et al., 2016). Platform leadership emphasizes the symbiotic and co-prosperous relationship among employees, leaders, and organizations. It emphasizes the aggregation of organizational resources and the improvement of employees’ capabilities. Based on contingency theory and resource-based view (RBV), this study argues that platform leadership places more emphasis on shaping organizational learning capabilities in the knowledge economy, which is conducive to the formation of organizational dual learning capabilities and thus brings sustainable competitive advantages to the enterprise. However, whether platform leadership is in line with the current competitive needs and whether it can bring a sustainable competitive advantage to the enterprise is yet to be verified. Therefore, this study explores the mechanism of platform leadership on enterprises’ sustainable competitive advantage through a questionnaire survey. It reveals the mediating role of ambidextrous learning in this process.

## Literature Review and Hypotheses

### Sustainable Competitive Advantage

Competitive advantage is a source of additional profits for enterprises. However, high profits will also attract competitors in the market to imitate enterprise behaviors and weaken the competitive advantage of enterprises. The combination of unique resources and internal and external environment brings some specific competitive advantages with high barriers to imitation for the company. Due to its uniqueness, this kind of competitive advantage can contribute additional profits to the company in the long term (Prabowo et al., 2021). Sustainable competitive advantage refers to the competitive advantage that existing and potential competitors cannot copy (Barney, 1991; Yang et al., 2021). RBV posits that enterprises have different tangible and intangible resources, some of which can be transformed into unique capabilities. These unique resources and capabilities are the source of lasting competitive advantage of enterprises. Enterprises with heterogeneous and incompletely transferable resources can better meet consumer demand, participate in market competition, create greater economic value, and thus establish sustainable competitive advantage (Oliver, 1997; Davcik and Sharma, 2016). Compared with general competitive advantage, sustainable competitive advantage lays more emphasis on enterprise resources’ inimitability and non-substitutability, which results in the sustainability of competitive advantage and a long-term positive impact on the firm (Khouroh et al., 2020). The studies conducted on the sustainable competitive advantage mainly started from the organizational level, such as knowledge acquisition (Asimakopoulos et al., 2020; Yang et al., 2021), organizational learning (Yang et al., 2021), dynamic capability (Khouroh et al., 2020; Prabowo et al., 2021) and so on. However, questions about how these organizational-level capabilities are acquired, and which influencing factors have an impact on these capabilities need further discussion.

### Platform Leadership

With the rapid development of the knowledge economy, enterprises face increasingly intensified market competition and a rapidly changing market environment. “Decentralization” has become an important development trend of organizations (Uhl-Bien et al., 2007). Organizational management pays more attention to employee’s self-realization needs and hopes to realize the flattening, coordination, flexibility, and decentralization of management (Xin et al., 2020). Management should focus on maintaining the employee–leader relationship and pay attention to the common development among employees, leaders, and organizations. Accordingly, the new type of leader not only focuses on the success of his or her career, but also pays more attention to enlarging the common platform with employees and constantly expands the common platform with employees (Sang et al., 2010). Moreover, Hao et al. (2021) clarified the specific concept of platform leadership and provided a way to measure it based on previous research. The literature indicates that platform leadership should promote the common growth of employees, leaders, and organizations by building a common career platform on the basis of stimulating employees’ potential (Hao, 2016; Hao et al., 2021). Accordingly, it should provide a measurement and dimensional division of platform leaders, including “tolerance,” “charisma,” “revolution planning,” “platform building,” “platform optimization,” and “mutual growth” (Hao et al., 2021). Previous empirical studies only focused on the impact of platform leadership at the employee level (Hao et al., 2021). However, the influence of platform leadership may go far beyond that.

Contingency theory states the importance of adapting to the internal and external environments of the organization, then choosing the appropriate management model and style to the different conditions and environments (Luthans and Stewart, 1977). The contingency theory of leadership states that no one leadership style can fit all organizations and environments, and leaders should adapt to changes in the external environment (Ruekert et al., 1985; Kerr et al., 2016). Management styles adapt to the organization and environment will be conducive to the improvement of team efficacy, team performance, and even enabling strategic alignment (Karim et al., 2016; Williams et al., 2017; McAdam et al., 2019). With the development of knowledge economy and the gradual flattening of organizations, platform leadership emphasizes the common development among employees, leaders, and organizations, to obtain or produce more high-quality resources—a leadership model that meets the requirements of the environment and organizations (Michael, 2012; Xin et al., 2020; Hao et al., 2021). The positive effect of platform leadership on organizational development and team performance is predictable.

Employees pay attention to the organization’s evaluation of their contributions and support for their development, and this attention forms a comprehensive feeling called perceived organizational support (Eisenberger et al., 1986). Organizational support theory suggests that employees who perceive organizational or team support will timely give positive signals to the organization, be enthusiastic about their work, display positive work attitudes and behaviors that are consistent with corporate development requirements, and positively influence employee and team performance (Rhoades and Eisenberger, 2002; Hui et al., 2007; Lavelle et al., 2009; Zagencryk et al., 2010). The characteristics of platform leadership, such as inclusiveness, platform building, platform optimization, and common growth, fully reflect the platform leaders’ attention to the organization and employees. Thus, platform leadership provides a prerequisite for employees to repay the team and contribute to the platform and organization.

Additionally, according to the RBV theory, a non-negligible relationship exists between organizational resources and sustainable competitiveness. Rose et al. (2010) pointed out that organizational resources include all the assets that an enterprise can control to implement its strategies and improve its efficiency. Moreover, the sustainable competitive advantage depends on the valuable and scarce resources that cannot be completely imitated or replaced by others. The employee support provided by platform leadership will enhance employees’ enthusiasm for work and promote the generation of work behaviors that meet the corporate development requirements. Besides, according to contingency theory, leadership that matches the environment will have a long-term, continuous and diffuse influence on organizations (McAdam et al., 2019). In other words, the positive impact of platform leadership on enterprises is not temporary. Platform leadership creates continuously valuable, scarce, and irreplaceable organizational resources for the company and promotes the generation of a sustainable competitive advantage for the organization. Thus, the platform leadership approach agrees with both the external environment matching, as evidenced by the power change management theory, and the employee development support advocated by the organizational support theory, which brings valuable and irreplaceable organizational resources to the company, thus enhancing the sustainable competitive advantage of the company.

Accordingly, this paper proposes the following hypothesis:

*H1:* Platform leadership positively impacts sustainable competitive advantage.

### Ambidextrous Learning

Organizational learning is an adaptive process of organizations to the external environment (March and Simon, 1958); it is also an important source of firm’s innovative advantage (Ge et al., 2016). March and Simon (1958) further interpreted the internal mechanism of organizational learning and proposed the concept of ambidextrous learning; that is, the organization pursues both exploratory and exploitative learning (Yang et al., 2012, 2021). Exploratory learning refers to the learning of product and process development skills that are completely new to the company’s existing experience and the ability to collect, learn, and research new knowledge that is different from the existing knowledge accumulation (Noni and Apa, 2015; Zhou et al., 2021). This type of learning acquires relevant knowledge and resources different from the organization’s existing knowledge accumulation, and the organization must obtain the knowledge through communication and cooperation with relevant external organizations (Zhu et al., 2014). Moreover, exploratory learning has strong uncertainty (Xie et al., 2014). Meanwhile, exploitative learning is the organization’s restructuring of information, resources, and knowledge based on the existing knowledge base (Noni and Apa, 2015; Huang et al., 2020). It emphasizes slow change and innovation in an existing product or knowledge domain (Li et al., 2013) and pays attention to efficiency, refinement, and implementation (Xie et al., 2014; Zhou et al., 2021).

Exploratory and exploitative learning are compatible and mutually reinforcing (Huang et al., 2020; Yang et al., 2021). In terms of the correspondence between the type of knowledge and organizational learning, exploratory learning is related to the knowledge acquired by the firm that is unfamiliar, future, and foreign (Yang et al., 2021; Zhou et al., 2021). Conversely, exploitative learning is associated with known, existing, and local knowledge (Niebles et al., 2008; Huang et al., 2020; Yang et al., 2021).

Platform leadership emphasizes support, understanding, and tolerance for employees; it does not mind the occasional mistakes in work and learning and gives employees and the organization more opportunities for trial (Hao et al., 2021). A more inclusive and relaxed work environment helps stimulate the creativity of employees and teams (Carmeli et al., 2010) and drives them to collect, learn, research new knowledge, and develop new competencies. Besides, platform leadership focuses on platform building and mutual growth, which strengthens communication and learning with those outside the organization (Sang et al., 2010; Hao et al., 2021). Then, it creates good conditions for teams and organizations to learn new knowledge, which in turn plays a positive role in organizational exploratory learning.

Simultaneously, platform leaders pay considerable attention to the growth of the organization and employees, give their subordinates sufficient space to complete the learning of intra-organizational knowledge, actively promote intra-organizational common knowledge, and are willing to create a good learning atmosphere within the company (Hao et al., 2021). Learning-oriented leadership behavior will improve organizational learning (Slater and Narver, 1995; Baker and Sinkula, 1999), and platform leaders’ encouragement of intra-organizational knowledge learning and exchange will also play a positive role in organizational exploitative learning.

Accordingly, this paper proposes the following hypothesis:

*H2:* Platform leadership positively affects exploratory learning.

*H3:* Platform leadership positively affects exploitative learning.

The role of ambidextrous learning for competitive advantage has been confirmed by many researchers (Sijabat et al., 2020; Wang and Fang, 2021). In related studies, exploratory learning and exploitative learning are always used to explain the mechanism of the effects of certain organizational capabilities on sustainable competitive advantage (Yang and Wang, 2020; Yang et al., 2021). In a complex, competitive environment, organizations should constantly learn to adapt to the challenges posed by the external environment (Yang et al., 2004). In cultivating a sustainable competitive advantage, organizational learning ability is an essential part. Therefore, the leadership model that can promote the improvement of organizational learning ability is vital.

Exploratory learning emphasizes acquiring relevant knowledge and information in new fields, and knowledge in relevant fields affects the firm’s recognition and perception of opportunities (Ge et al., 2016). The continuously accumulated experience and knowledge help enrich the variety of organizational knowledge resources. Moreover, the sufficient number and type of knowledge resources are important inducements for the organization to form irreplaceable resources and capabilities, which help the firm to continuously improve its competitive ability (Yang et al., 2021). Exploratory learning affects enterprises’ ability to adapt to the new environment and decision-making speed and emphasizes experiment and innovation (Li et al., 2013; Sijabat et al., 2020; Zhou et al., 2021). It breaks existing learning paths and practices, promotes the reorganization and reconstruction of resources, and improves organizational environment adaptability. It also develops dynamic capabilities and forms sustainable competitive advantages.

According to the contingency theory, the appropriate management adapted to the internal and external environments has a positive impact on organizational behavior (Luthans and Stewart, 1977; McAdam et al., 2019). Platform leaders, as the suitable leaders for the current competitive environment, focus on platform building and platform optimization, drive the exchange of knowledge outside the organization and create an inclusive innovation environment. All are important conditions for the formation of organizational exploratory learning capability and an important guarantee for the formation of sustainable competitive capability.

Exploitative learning emphasizes the understanding and usage of existing knowledge and resources, and by expanding the content and depth of knowledge resources, it helps organizations enhance dynamic capabilities and sustainable competitiveness (Zollo and Winter, 2002; Wang and Fang, 2021; Zhou et al., 2021). Repeatedly learning and improving existing knowledge and resources help enterprises understand market knowledge and current competitive situation. These methods also improve their ability to obtain and take advantage of timely opportunities. Simultaneously, through the use of knowledge and experience accumulated continuously, enterprises form and consolidate their own resource advantages and create sustainable competitive ability.

Organizational support theory states that employees who perceive organizational support will display positive work attitudes and behaviors that are consistent with corporate development requirements (Zagencryk et al., 2010; Matusik et al., 2021). Platform leadership’s encouragement of knowledge flow within enterprises will active employee learning organizational knowledge, then help improve the exploitative learning ability of organizations, thus contributing to the formation of the sustainable competitive capability of enterprises.

Accordingly, this paper proposes the following hypothesis:

*H4:* The effect of platform leadership on a sustainable competitive advantage is mediated by exploratory learning.

*H5:* The effect of platform leadership on a sustainable competitive advantage is mediated by exploitative learning.

**Figure 1:**
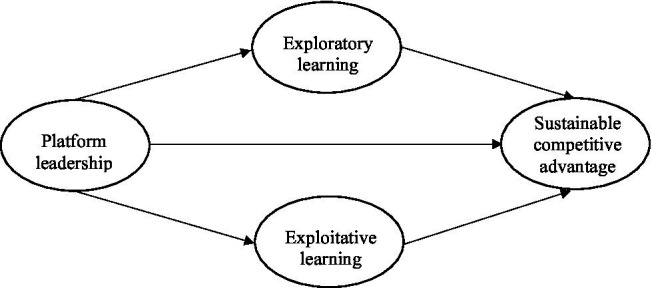
Research framework.

Based on the above hypotheses, this study proposes the following research framework Figure 1.

## Research Methods

### Data Collection and Sample

To verify the hypotheses, we applied a questionnaire survey to collect data. The survey was conducted between April 1 and August 30, 2021. Most questionnaires used mature scales. To ensure the validity of data and avoid ambiguity caused by terminology, expression, and other reasons, the research group conducted a preliminary survey before the formal survey. Fifteen managers, EMBA, and MBA students who have a long-term cooperative relationship with the research group were selected to conduct a preliminary questionnaire test. The questionnaire was improved according to the testers’ opinion to ensure that the respondents could fully understand the meaning of each item. Pre-survey data were excluded from the final data.

We obtained the final data from China, which is a vast country that encompasses various regions. Different regions have different cultures, government policies, and locational conditions. To reduce the influences of these situational factors on the research results, we strategically selected the northeast region for our research. As the research focuses on platform leadership and ambidextrous learning, respondents were required to have some knowledge of the company’s innovation capability and competitiveness. Thus, we chose middle-level and above managers in relevant enterprises.

To ensure the integrity and reliability of the data, the research adopted a survey method combining online directional distribution and offline field distribution to collect the data. Finally, under the coordination of alumni and relevant government departments, 289 questionnaires were collected, and 23 random and incomplete questionnaires were excluded. Finally, 266 valid questionnaires were obtained. The detailed characteristics of sampled firms are shown in Table 1.

**Table 1:** Profile of sampled firms.

Characteristics of Firms		Frequency	Percentage (%)
Industry characteristics (Industry)	high-tech industries	120	45.11
	other industries	146	54.89
Firm age	1–5 years	16	6.02
(Age)	6–10 years	56	21.05
	11–20 years	128	48.12
	over 20 years	66	24.81
Number of employees	less than 100	49	18.42
(Size)	100–500	141	53.01
	more than 500	76	28.57
Ownership	State owned	82	30.83
(Ownership)	Privately owned	129	48.5
	Foreign owned	35	13.16
	Sino-foreign joint	20	7.52

### Variables and Measures

This section introduces the main research variable and control variables. It also presents descriptive statistics and the correlation matrix for all variables (Table 2).

**Table 2:** Summary statistics and correlation matrix.

Variables	Mean	SD	1	2	3	4	5	6	7
SCA	5.113	1.057							
EE	59.318	0.971	0.394^**^						
EY	4.956	0.982	0.364^**^	0.289^**^					
PL	5.239	0.674	0.400^**^	0.421^**^	0.405^**^				
Age	2.92	0.834	0.021	0.013	0.046	0.184^**^			
Ownership	1.97	0.862	−0.017	0.061	−0.009	−0.001	−0.266^*^		
Industry	0.45	0.499	0.071	0.106	0.05	−0.045	−0.091	−0.130^*^	
Size	2.1	0.679	0.018	−0.061	0.001	0.047	0.395^**^	−0.105	−0.203^**^

* *p* ≤ 0.05;

** *p* ≤ 0.01.

#### Main Research Variable

To ensure the reliability and validity of the questionnaire, this study mainly referred to relevant scales published in authoritative journals to measure the main variables. We revised and improved the scales by using trial investigation and discussion with experts. The questionnaire consisted of four parts, including three construct measurements and control variable measurement. All measures were adapted from existing scales found in previous studies. The measurement of the platform leadership (PL) is adopted from Hao et al. (2021). Moreover, the measurement of exploratory learning (EY) and exploitative learning (EE) was prepared and adopted from Atuahene-Gima and Murray (2007) and Chung et al. (2015). Lastly, the measurement of the sustainable competitive advantage (SCA) mainly referred to Yang and Wang (2020) and Yang et al. (2021). This study conducted a tick-the-box survey. All the items in the construct measurements were measured using a 7-point Likert scale ranging from “strongly disagree = 1” to “strongly agree = 7.”

#### Control Variables

Besides, previous studies have suggested that a firm’s ambidextrous learning and the competitive advantage may be influenced by firm age (*age*), firm size (*size*), ownership (*ownership*), and industry characteristics (Camisón and Villar-López, 2014; Ma and Wu, 2020; Yang and Wang, 2020). For industry characteristics (*industry*), relevant research should compare high-tech and other industries (Ge et al., 2016). Accordingly, in terms of industry characteristics, industries with high technology content, such as software, computer, network, telecommunications, electronics, communications, polymer, chemical, and biopharmaceutical, are divided into high-tech industries and set as 1, whereas other industries are set as 0. We included these control variables in the study, and the results of relevant variables are shown in Tables 1 and 2.

## Results

### Measurement Model Analysis

We assessed the unidimensionality of the latent variables using confirmatory factor analysis. The model fit indices were as follows: *χ*^2^ = 870.937, degree of freedom (df) = 743, *p* < 0.001, *χ*^2^/df = 1.172, comparative fit index (CFI) = 0.980, Tucker–Lewis index (TLI) = 0.978, incremental fit index (IFI) = 0.980, root mean square error of approximation (RMSEA) = 0.025, thus meeting the requirements of the cutoff values (Hair et al., 2013).

We also measured the convergent and discriminant validity of the constructs. The convergent validity of the constructs was assessed using composite reliability (CR) and average variance extracted (AVE) values (Fornell and Larcker, 1981; Hair et al., 2013). Table 3 show each construct’s Cronbach’s α and CR values. Platform leadership is composed of six constructs (*Tolerance*, *Charisma*, *Platform Building*, *Revolution Planning*, *Platform Optimization*, *and Mutual Growth*), each of which contains 3–5 measurement items (Hao et al., 2021), and each construct’s Cronbach’s *α* and CR values are calculated separately. Cronbach’s *α* values of all the constructs ranged from 0.742 to 0.928, exceeding the recommended minimum standard of 0.70 (Fornell and Larcker, 1981). All factor loadings were higher than 0.65, indicating strong convergent validity (Anderson, 1987). Moreover, all of the CR values were greater than 0.850, which is greater than the minimum acceptable value of 0.7. Furthermore, the AVE values exceed the suggested standard of 0.50, which ultimately confirms the necessary reliability and convergent validity.

**Table 3:** Convergent and discriminant validity.

Constructs	1	2	3	4	5	6	7	8	9
SCA	**0.730**								
EE	0.568	**0.731**							
EY	0.608	0.615	**0.727**						
MG	0.664	0.462	0.554	**0.789**					
PO	0.655	0.512	0.490	0.604	**0.797**				
RP	0.496	0.656	0.523	0.555	0.526	**0.841**			
PB	0.575	0.567	0.541	0.532	0.482	0.556	**0.776**		
CH	0.681	0.635	0.532	0.626	0.582	0.584	0.709	**0.745**	
TO	0.609	0.515	0.555	0.525	0.399	0.409	0.422	0.706	**0.729**
Cronbach’s α	0.928	0.889	0.880	0.827	0.820	0.742	0.826	0.873	0.856
AVE	0.679	0.649	0.608	0.622	0.635	0.707	0.601	0.556	0.531
CR	0.927	0.902	0.886	0.868	0.874	0.878	0.858	0.862	0.850

Bold values: Square root of AVE for each construct.

Fornell and Larcker (1981) suggested that we used AVE to measure discriminant validity. Table 3 demonstrates that the square root of the AVE for each construct (highlighted in bold on the diagonal) is higher than the correlation between any pair of distinct constructs, providing evidence of discriminant validity.

### Common Method Variance

Common method variance (CMV) was a concern in this study, as each questionnaire was finished by a single respondent (Podsakoff et al., 2003). We tried reducing the potential influence of CMV by carefully selecting scale items and separating them within the lengthy questionnaire. Then, we used two methods to check for CMV. First, Harman’s single-factor test was used to examine the effect of homology bias (Podsakoff et al., 2003). The results showed that the variance explanation degree of the first factor was 31.36%, which is lower than 50%, indicating that CMV was not a serious concern. Second, we completed the correlation coefficient test of latent variables (Table 3). The absolute value of correlation coefficient between latent variables was less than 0.709, far less than 0.9, indicating no significant common variance deviation in the research data (Podsakoff et al., 2003). The analysis indicates that CMV does not pose any risk or concerns for the results of this study.

### Hypothesis Testing

First, we employed multiple linear regression, using SPSS 24.0, to test the relationship between platform leadership and a sustainable competitive advantage (H1), the relationship between platform leadership and exploratory learning (H2), and the relationship between platform leadership and exploitative learning (H3). In addition, we performed collinearity tests. The results show that the maximum variance inflation factor value is 1.309, below the cutoff point of 4.0, indicating that the research results are not affected by multicollinearity issue (Pallant, 2016). The results are shown in Table 4.

**Table 4:** Results of multiple linear regression.

Variables	SCA		EY		EE	
	Model 1	Model 2	Model 3	Model 4	Model 5	Model 6
*Control Variables*						
Age	0.017	−0.069	0.058	−0.027	0.069	−0.021
ownership	0.001	−0.019	0.013	−0.006	0.089	0.068
industry	0.079	0.090	0.055	0.067	0.113	0.124
Size	0.027	0.042	−0.009	0.006	−0.056	−0.040
*Main Research Variable*						
PL		0.415^***^		0.413^***^		0.432^***^
*R* ^2^	0.006	0.172	0.005	0.169	0.022	0.201
Adjusted *R*^2^	−0.009	0.156	−0.010	0.154	0.007	0.186
*F*	0.423	10.818^***^	0.352	10.611^***^	1.447	13.095^***^

*** *p* ≤ 0.001.

The independent variables in model 1 contained only control variables, and the dependent variable was a sustainable competitive advantage. Model 2 adds the variable of platform leadership. The empirical analysis results show that platform leadership had a significant positive correlation with a competitive advantage (*β* = 0.415, *p* < 0.001), thereby supporting H1. The dependent variable in Models 3 and 4 is exploratory learning. Model 4 shows that platform leadership has a significant positive influence on exploratory learning (*β* = 0.413, *p* < 0.001). In the same way, the results in Model 6 show that platform leadership has a significant positive influence on exploitative learning (*β* = 0.432, *p* < 0.001), supporting H2 and H3.

Then, we employed the bootstrap method to test the mediation effects. Using the SPSS macro program Process3.4, we demonstrate the mediating role of exploratory and exploitative learning based on 5,000 iterations at the 95% confidence interval (CI; see Table 5).

**Table 5:** Mediating effect result (*N* = 266).

Effect type	Path relationship	Effect value	*SE*	95% CI	*t*-value	*p*-value
Total effect	PL → SCA	0.651	0.090	[0.473, 0.829]	7.216	0.000
Direct effect	PL → SCA	0.356	0.100	[0.159, 0.553]	3.559	0.000
Total indirect effect	PL → SCA	0.295	0.073	[0.167, 0.459]		
Indirect effect1	PL → EY → SCA	0.131	0.055	[0.044, 0.259]		
Indirect effect2	PL → EE → SCA	0.163	0.059	[0.062, 0.298]		

**Figure 2:**
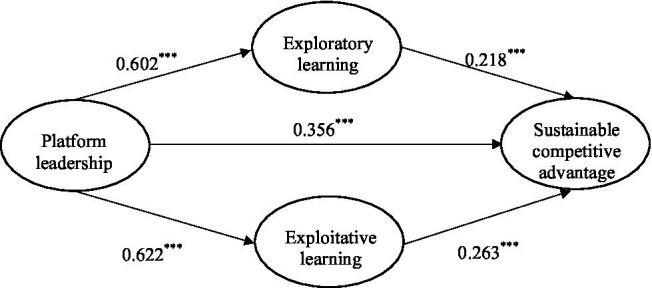
Hypotheses testing. ***p ≤ 0.001.

In the mediating effect test of exploratory learning and exploitative learning, the total effect of platform leadership on a sustainable competitive advantage is 0.651 at 95% CI [0.473, 0.829], not including 0. The direct effect of platform leadership on a sustainable competitive advantage is 0.356 at 95% CI [0.159, 0.553]. The indirect effect of exploratory learning (0.131 at 95% CI [0.044, 0.259]) is significant, supporting H4. Similarly, the indirect effect of exploitative learning (0.163 at 95% CI [0.062, 0.298]) is significant, thus supporting H5 (Figure 2). Items for constructs are shown in Table 6.

**Table 6:** Measurement of scale.

Constructs	Items
Tolerance (TO)	TO1: My leader does not mind if his subordinates are better than himself in some aspects
	TO2: My leader does not mind occasional mistakes in his subordinates’ work
	TO3: My leader does not mind sharing honors and opportunities with his subordinates
	TO4: My leader does not mind and often encourages his subordinates to give him advice
	TO5: My leader respects his subordinates’ differences in personalities and abilities
Charisma (CH)	CH1: My leader always stays positive in good times and bad
	CH2: My leader can put himself in his subordinates’ shoes
	CH3: My leader does not give up when things get tough
	CH4: My leader can make decisions quickly and accurately when encountering emergencies or important cases
	CH5: My leader can deal with problems objectively and fairly
Platform Building (PB)	PB1: My leader has full confidence in his subordinates’ work ability and personal character
	PB2: My leader believes that the interests of his subordinates agree with those of the organization
	PB3: My leader is committed to continuous improvement of existing organizational systems
	PB4: My leader has sufficient socio-economic resources to help the organization achieve its goals
Revolution Planning (RP)	RP1: My leader has a long-term plan for developing the company/team
	RP2: My leader can quickly identify and summarize the essence of problems
	RP3: My leader can clearly set and describe the vision of the organization
	Platform Optimization (*α* = 0.856)
Platform Optimization (PO)	PO1: My leader is good at motivating subordinates to pursue higher goals
	PO2: My leader encourages subordinates to embrace and learn all the knowledge beneficial to organizational development and personal improvement
	PO3: My leader encourages subordinates to constantly seek new ideas and approaches in solving problems
	PO4: My leader communicates frequently and proactively with subordinates emotionally
Mutual Growth (MG)	MG1: My leader often pays attention to their growth and gives his subordinates guidance and education
	MG2: My leader continues to learn advanced professional knowledge and leadership skills
	MG3: My leader creates opportunities to fully empower subordinates to take charge of a project
	MG4: My leader often communicates with subordinates about new technologies and knowledge to help them grow
Exploratory Learning (EY)	EY1: In information search, we focused on mastering project strategies that involved experimentation and high market risks
	EY2: We preferred to collect information with no identifiable strategic market should ensure experimentation in the project
	EY3: Our aim was to acquire knowledge to develop a project that led us into new areas of learning, such as new markets and technological areas
	EY4: We collected novel information and ideas that went beyond our current market and technological experiences
	EY5: We collect new information that forced us to learn new things in the product development project
Exploitation Learning (EE)	EE1: We search for information to refine common methods and ideas in solving problems in the project
	EE2: Search for ideas and information that we can implement well to ensure productivity rather than those ideas that could lead to implementation mistakes in the project and in the marketplace
	EE3: We searched for usual and proven methods and solutions to product development problems
	EE4: We used information acquisition methods (e.g., survey of current customers and competitors) that helped us understand and update the firm’s current project and market experiences
	EE5: We emphasized the use of knowledge related to our existing project experience
Sustainable Competitive Advantage (SCA)	SCA1: The quality of the products or services that my firm offers is better than that of the competitor’s products or services
	SCA2: My firm is more capable of R&D than the competitors
	SCA3: My firm has better managerial capability than the competitors
	SCA4: My firm’s profitability is better
	SCA5: The corporate image of my firm is better than that of the competitors
	SCA6: The competitors are difficult to take the place of my firm’s competitive advantage

## Conclusion

This paper combines the RBV theory with the contingency theory to validate the positive impact of platform leadership on a sustainable competitive advantage through data. Additionally, this paper verifies the mediating role of ambidextrous learning, in which platform leadership contributes to the improvement of exploratory and exploitative learning in the organization, and ultimately to the improvement of sustainable competitiveness. In this context, our study contributes to both theory and practice.

### Discussion

With the advent of the knowledge-based economy, organizations have been developing to be more suitable for information exchange and learning to meet the increasingly competitive marketplace (Meisel and Fearon, 1999; Yang et al., 2004). In this context, the importance of leadership in business has been widely debated in academia, and de-leadership was once an accepted development approach (Uhl-Bien et al., 2007; Avolio et al., 2009). However, this paper concludes that choosing the right leadership model is still the key to sustainable competitiveness even in a highly competitive market. Platform leadership is a leadership model that focuses on the tripartite development of platform, leader, and employees; it has a significant positive effect on the sustainable competitiveness of an enterprise. For enterprises, platform leadership, with its tolerance, unique personal charisma, emphasis on platform building, platform optimization, revolution planning, and mutual growth (Hao et al., 2021), will be conducive to the formation of irreplaceable organizational resources, generate long-term positive effects for the organization and ultimately contribute to the formation of sustainable competitiveness of the enterprise.

Additionally, this paper presents and tests for the first time the positive contribution of platform leadership to ambidextrous learning. Platform leadership focuses on employee development and platform optimization and plays a positive role in promoting the flow of organizational knowledge within the company, which will promote the development of the organization’s ability to leverage learning. Simultaneously, studies have evaluated the positive effect of platform leadership on employee innovation behavior (Hao et al., 2021). This shows that platform leadership, with its tolerance leading to employee learning and innovation, and focusing on the interaction of information from the platform to the outside of the organization, lays a good foundation for exploratory learning.

Finally, this study examines the mediating role of ambidextrous learning in the relationship between platform leadership and sustainable competitiveness of the firm. The results of this study indicate that platform leadership ultimately contributes to the improvement of sustainable competitiveness of the firm by driving the improvement of organizational exploratory and exploitative learning capabilities. Additionally, the findings show that ambidextrous learning is an incomplete mediating role. These findings suggest that platform leadership leads to sustainable competitiveness by enhancing organizational ambidextrous learning capabilities and other means. Therefore, the paths of platform leadership for a sustainable competitive advantage of enterprises are more complex than expected and can be explored more deeply by future research.

### Theoretical Contributions

This study has three theoretical contributions. First, it complements the research on the impact of platform leadership on organizational behavior and capability, which is a continuation and improvement of leadership theory in the current competitive environment. Previous studies on platform leadership have mostly focused on theoretical exploration and dimensional model construction (Michael, 2012; Hao, 2016; Xin et al., 2020; Hao et al., 2021). In particular, related empirical studies focus on the role of platform leadership on employees’ innovative behavior (Hao et al., 2021). However, compared to previous studies, this present study breaks through the research related to the impact of platform leadership from the level of individual behavior to the level of organizational behavior and organizational capability, which is an important addition to the research on platform leadership and leadership theory.

Second, this study has expanded the application of contingency theory in the era of knowledge economy, which is an important addition of contingency theory. The contingency theory proposes that management models must respond and change according to the organization’s internal and external conditions to gain management advantages and improve team performance (Luthans and Stewart, 1977; Karim et al., 2016; Williams et al., 2017). The recent studies applied contingency theory to leadership mainly focus on using a bottom-up approach to lead employees, respond to changes in the external environment, and facilitate the achievement of organizational goals (Morris et al., 2005; Owens and Hekman, 2012; Javed et al., 2020; Ziegert et al., 2021). This study’s results show that platform leadership, as a leadership model that can balance the development of employees, platforms, and leaders, can lead to sustainable competitive advantage and contribute to the improvement of organizational ambidextrous learning capabilities, which is a leadership model adapted to the current knowledge economy environment. Simultaneously, the results of this study once again prove that a leadership model that adapts to the internal and external environment of the organization has a positive effect on the development of the company, refuting the useless leadership view in the knowledge economy. Moreover, it is an important complement to and development of the contingency theory and leadership theory.

Third, this study combines RBV theory and contingency theory to further interpret the formation of a sustainable competitive advantage. In a knowledge-based economy, academic and practical communities have paid less attention to leadership than before, and the role of leadership for firms has been questioned by the proliferation of ideas, such as decentralization and de-leadership (Uhl-Bien et al., 2007; Avolio et al., 2009). The studies conducted on the sustainable competitive advantage of firms have also mostly started from the organizational level (Asimakopoulos et al., 2020; Khouroh et al., 2020; Prabowo et al., 2021; Yang et al., 2021). This paper shows that sustainable competitive advantage can be built from the individual level. Platform leadership, as the appropriate leadership at present, can also be regarded as an important enterprise resource. The results state that platform leadership can promote dual learning capabilities and the generation of non-substitutable resources, thus increasing the firm’s sustainable competitive advantage. This study extends and develops the RBV theory by exploring the causes of a sustainable competitive advantage from a new perspective of platform leadership.

### Managerial Implications

The findings of this study also provide important insights for corporate development. First, companies should attach importance to the vital role of platform leadership to lay a foundation for the formation of a sustainable competitive advantage. Compared with other leadership, platform leadership can balance the development of employees, platforms, and leaders better. In the operation practice, the stable, harmonious, and mutually supportive tripartite relationship among employees, leaders, and platforms will provide driving force for the development of organizations and have a positive impact on sustainable competitive advantage. Companies should recognize the long-term impact of platform leadership on their competitive advantage. Institutions ought to be adopted to encourage the steady formation and development of platform leadership. Enterprises can select the right leaders according to the connotation of platform leadership or encourage leaders to transform into platform leaders by the enterprise system. Additionally, companies can give platform leaders the appropriately broad authority to ensure that the positive effects of platform leadership on the organization can be successfully implemented over the long term.

Second, research has demonstrated that organizational learning can be improved and developed by choosing the right leadership model. It is a consensus among academics and practitioners that improving organizational learning capability leads to a sustainable competitive advantage. This study shows that the right type of leadership can lead to the improvement of organizational learning ability. Through the joint of employees, platforms, and leaders, platform leaders will extend its contribution to the enterprise from the employee level to the organizational level. Platform leadership has a significant positive impact on exploratory learning and exploitative learning, which in turn promotes the formation of unique resources that are difficult to replicate. Therefore, relevant enterprises can start with the key dimensions of platform leadership model, then gradually promote the formation of good organizational learning habits. Especially for those companies in fast-growing industries, the rapid changes in the external environment that companies face rely heavily on organizational learning capabilities. The selection of platform leadership model will facilitate the multi-value of employees, leaders, and the platform, forming an organizational atmosphere of continuous learning. Continuous organizational learning capability will provide a constant competitive advantage and the long-term development of enterprises.

### Limitations and Future Directions

This study has following limitations that have implications for future research. First, as one of the first papers to study the effect of platform leadership on sustainable competitive advantage, our research views platform leadership concept as a whole. Future research could adopt the same research method to explore the subdimensions of platform leadership, such as “tolerance,” “charisma,” “revolution planning,” “platform building,” “platform optimization,” and “mutual growth” (Hao et al., 2021). Second, the results of this study indicate that ambidextrous learning is an incomplete mediating role, which means the paths of platform leadership for sustainable competitive advantage are more complex. Future research could choose other perspectives to further improve the mechanism of platform leadership on sustainable competitive advantage. Third, this paper chooses questionnaire method to complete this research, and all our respondents are Chinese residents. According to the contingency theory, the internal and external environments of the organization are important factors in choosing the appropriate leadership model (Luthans and Stewart, 1977; Williams et al., 2017). China’s economy has grown rapidly in recent years and its business environment is quite representative in the international market. However, in some economically underdeveloped areas, the applicability of the findings of this paper remains to be considered. Future studies may consider regions with different economic development to further examine the complex effects of regional economic development on the findings of this paper.

## Data Availability Statement

The raw data supporting the conclusions of this article will be made available by the authors, without undue reservation.

## Author Contributions

XY: conceptualization, funding acquisition, project administration, methodology, investigation, writing—review, and supervision. RJ: investigation, validation, and writing—original draft. CZ: formal analysis, methodology, and writing—review and editing. All authors contributed to the article and approved the submitted version.

## Funding

This research was funded by the National Social Science Foundation of China (Grant No. 18BGL032), Soft Science Foundation of Sichuan Province (Grant Nos. 2019JDR0190 and 2021JDR0109), Soft Science Foundation of Chengdu (Grant No. 2019-RK00-00402-ZF), the Fundamental Research Funds for the Central Universities (Grant Nos. 2021ZY SX20 and 2021ZZ006), and Sichuan university (Grant No. 2021CXC23).

## Conflict of Interest

The authors declare that the research was conducted in the absence of any commercial or financial relationships that could be construed as a potential conflict of interest.

## Publisher’s Note

All claims expressed in this article are solely those of the authors and do not necessarily represent those of their affiliated organizations, or those of the publisher, the editors and the reviewers. Any product that may be evaluated in this article, or claim that may be made by its manufacturer, is not guaranteed or endorsed by the publisher.
